# Young people’s views about consenting to data linkage: findings from the PEARL qualitative study

**DOI:** 10.1186/s12874-016-0132-4

**Published:** 2016-03-21

**Authors:** Suzanne Audrey, Lindsey Brown, Rona Campbell, Andy Boyd, John Macleod

**Affiliations:** School of Social and Community Medicine, University of Bristol, Canynge Hall, Whatley Road, Bristol, BS8 2PS UK; ᅟ, ᅟ, Bristol, UK

**Keywords:** Data linkage, Consent, Young people, Qualitative research, ALSPAC

## Abstract

**Background:**

Electronic administrative data exist in several domains which, if linked, are potentially useful for research. However, benefits from data linkage should be considered alongside risks such as the threat to privacy. Avon Longitudinal Study of Parents and Children (ALSPAC) is a birth cohort study. The Project to Enhance ALSPAC through Record Linkage (PEARL) was established to enrich the ALSPAC resource through linkage between ALSPAC participants and routine sources of health and social data. Qualitative research was incorporated in the PEARL study to examine participants’ views about data linkage and inform approaches to information sharing. This paper focusses on issues of consent.

**Methods:**

Digitally recorded interviews were conducted with 55 participants aged 17–19 years. Terms and processes relating to consent, anonymization and data linkage were explained to interviewees. Scenarios were used to prompt consideration of linking different sources of data, and whether consent should be requested. Interview recordings were fully transcribed. Thematic analysis was undertaken using the Framework approach.

**Results:**

Participant views on data linkage appeared to be most influenced by: considerations around the social sensitivity of the research question, and; the possibility of tangible health benefits in the public interest. Some participants appeared unsure about the effectiveness of anonymization, or did not always view effective anonymization as making consent unnecessary. This was related to notions of ownership of personal information and etiquette around asking permission for secondary use. Despite different consent procedures being explained, participants tended to equate consent with ‘opt-in’ consent through which participants are ‘asked’ if their data can be used for a specific study. Participants raising similar concerns came to differing conclusions about whether consent was needed. Views changed when presented with different scenarios, and were sometimes inconsistent.

**Conclusions:**

Findings from this study question the validity of ‘informed consent’ as a cornerstone of good governance, and the extent to which potential research participants understand different types of consent and what they are consenting, or not consenting, to. Pragmatic, imaginative and flexible approaches are needed if research using data linkage is to successfully realise its potential for public good without undermining public trust in the research process.

## Background

### Research ethics and consent

Evidence of the abuse suffered by human research ‘subjects’ during the Second World War prompted the development of frameworks for ethical medical research which were enshrined in the Nuremberg Code (1947) and the Helsinki Declaration (1964) [1, 2]. Local Research Ethics Committees were formed in the UK in early 1968 to regulate medical research and ensure adherence to these frameworks. Consent which is properly informed and freely given is central to research ethics [3], but the scandal at Alder Hey Hospital in England, where dead children’s organs had been retained for research without parental consent, highlighted the potential for unethical research practice [4]. In response, in 2001 the Department of Health developed the Research Governance Framework for England with the aim of providing a coherent and ethical framework for health research [5, 6]. As well as participants being fully informed about the risks and benefits of participating in research, consent must be freely given and is often considered a process through which the option of withdrawing consent at any time is incorporated into the consent procedure.

### Data linkage

Electronic administrative data, collected for purposes other than research but potentially useful for research, exist in several health and social domains. These data can enhance epidemiological research in several ways. In the case of established research study samples, data linkage can provide a means of cost efficient follow-up. Information available through linkage may be difficult to collect in other ways, such as participant self-report, or if self-reported may be subject to bias [7]. Data linkage can provide information that would otherwise be missing and can be used to inform analytic strategies to address the issue of missing data [8]. Linking information about the same individuals, from different data sources, can assist in establishing links between health and the environment, ‘lifestyle’ and other risk factors [9–12]. The knowledge gained is likely to increase with the quantity and quality of data made available.

However, benefits derived from linking personal data need to be considered alongside the risks. If ‘participation’ in linkage based research exposes individuals to risk of harm then the issue of their consent arises. The main objective risk related to such participation is the threat to privacy. Deductive disclosure remains a theoretical risk in research data sets unless all steps to remove uniqueness are taken. However in practice there are no examples of privacy breaches of this nature arising from epidemiological research that we are aware of. Individuals may also be unhappy about the use of information contained in ‘their’ records, for example, if they do not approve of the research topic or the possible use of the findings. Whether harm of this nature is substantial enough to require researchers to seek consent is contested. In general, research based on anonymised records does not legally require the consent of the individuals from whom the records are derived [13].

Data linkage typically involves identifiable records, at least at the point of linkage. In practice, privacy concerns may be largely overcome if anonymised linkage technologies [14, 15] or two-stage ‘split file’ approaches are used. In the case of the latter, a two-stage process is implemented: a trusted linkage agency uses personal identifiers to link data for the same individuals from different data-bases but without the agency having access to sensitive information, and; once the linkage is complete, personal identifiers are removed and sensitive information is restored to provide an anonymised dataset for researchers. Such datasets are more properly described as pseudonymised as a unique combination of variables within them may permit deductive identity disclosure. This consideration can be mitigated by further technical strategies (such as “k-anonymisation”) to remove uniqueness [16]. However, since these also reduce the information contained within the dataset, they may limit inferences that can be made from it.

### ALSPAC and PEARL

Avon Longitudinal Study of Parents and Children (ALSPAC) is the largest birth cohort with detailed biological and behavioural data from before birth to early adulthood in the world (http://www.alspac.bris.ac.uk). ALSPAC initially recruited 14,541 pregnant women resident in and around the City of Bristol, UK with expected dates of delivery 1st April 1991 to 31st December 1992. When the index children reached seven years of age, an attempt was made to bolster the initial sample with eligible cases who had failed to join the study during pregnancy. An additional 713 children were subsequently enrolled. The total sample size is therefore 15,247 pregnancies, resulting in 15,458 fetuses, of which 14,701 were alive at 1 year of age [17]. Since then, participants have been assessed, mainly through questionnaires and clinics and analysis of biological and genetic samples. The study website describes the resource in more depth (http://www.bris.ac.uk/alspac/researchers/data-access/data-dictionary/>).

The Project to Enhance ALSPAC through Record Linkage (PEARL) was established to obtain consent for, and establish mechanisms of, linkage between ALSPAC participants and routine sources of health and social data. The aim is to enrich the study database, enhance strategies to reduce the bias that may result from missing data and enable cost-efficient prospective follow-up in a way that minimises participant burden. Based on ethico-legal considerations and the requirements of the owners of routine records, alongside those related to etiquette and participant trust, a decision was made to seek explicit informed participant consent for linkage to a range of health and social administrative data held electronically. A postal consent campaign was launched which asked participants to record separate consent decisions for ALSPAC’s use of their health, education, economic and criminal routine records [18]. A total of 13,136 consent packs were sent out to ALSPAC participants: 3622 packs (28 %) had been returned by June 2013 of which 2754 (81.2 %) participants consented to all linkages, 584 (17.2 %) varied their consent decisions, and 55 (1.6 %) refused all linkages.

Qualitative research was incorporated in the larger PEARL study with the aim of examining participants’ perceptions of data linkage, their views on its advantages and disadvantages and a detailed exploration of any apprehensions about data linkage processes. The interviews formed part of ALSPACs work to ensure active participant involvement in the research process and, in this case, to shape the appropriate strategies and materials used in ALSPAC in relation to the study’s use of data linkage and participants’ routine records [19]. Findings from the qualitative study will also inform the wider public debate about record linkage. In this paper we focus specifically on the issue of consent.

## Methods

### Recruitment

The recruitment strategy was designed to maximise the study’s ability to interview participants from a range of health and social backgrounds and historical participation levels in ALSPAC. Initial sampling was random. To ensure that views were heard from a diverse range of participants, additional purposive sampling was based on random selection within sub-cohorts with one of three a-priori selected characteristics: i) low socio-economic status, ii) low participation history, and iii), whether the participant was a ‘care case’ (a study administration flag indicating that there was some sensitivity around contact due to health or family circumstances). Low socio-economic status was characterised as those living in areas amongst the two most deprived quintiles as indicated in the Index of Multiple Deprivation (IMD), a neighbourhood score compiled using census and local authority data. IMD status was determined through linking participants’ postal address to public domain IMD indexes. Participation history was characterised as either ‘engaged’ (participating at least once between 16 and 18 years of age), ‘partially engaged’ (some participation between >12 and <16 years of age, but none since then), ‘disengaged’ (last participation <12 years of age) or ‘eligible to participate’ (eligible to take part, but had never previously participated). A ‘tracing’ exercise was conducted to find up-to-date address details, including linking to the participants’ National Health Service (NHS) General Practitioner address.

All lists were checked by the ALSPAC family liaison team who removed a small number of names coded for whom health or family circumstances were such that it would have been inappropriate for an invitation to have been sent. A letter of invitation and a participant information leaflet were posted to 943 potential participants (194 sampled randomly and 749 purposively sampled) with a reply slip and stamped addressed envelope for them to return if they were interested in being interviewed. One postal reminder was sent. Those who returned a reply slip and expressed an interest were contacted by telephone by a researcher (LB) and face-to-face interviews arranged. Written consent was obtained from all participants prior to interview. All interviews were conducted before the PEARL consent campaign commenced. Ethical approval for the study was given by the ALSPAC Ethics and Law Committee (Reference number E200905). The Teenage Advisor Panel (TAP), made up of participants in the ALSPAC study, advised on study materials and communication for the study, and participated in the recruitment process for the study researcher/interviewer (LB).

### Interviews

Digitally audio-recorded interviews were conducted by one researcher (LB) and took place in the participant’s home, the University of Bristol or a public place chosen by the participant such as a coffee shop. Each participant was interviewed once on a one-to one basis. The topic guide was initially based on a review of the literature and discussions within the research team, and was refined as new areas of interest emerged during the first and subsequent interviews. Topics included: research using personal or sensitive information, record linkage, different types of consent, who should access records and for what purpose, privacy and confidentiality. At relevant stages during the interviews explanations were provided, aided by diagrams that were sketched by the researcher and/or participant, in relation to opt-in consent (potential participants are asked if they are willing to take part in a study and agree to do so) and opt-out consent (potential participants are informed about a study and, if they do not refuse, they are included in the study), data linkage processes, and anonymisation strategies. Following these more general topics, four scenarios (Table 1) were included to focus the discussion more specifically on linking different types or sources of data, whether consent should be requested, and if so what type of consent.

**Table 1:** Data linkage scenarios

Scenario 1: The Government is concerned about the number of teenage pregnancies in the South West. Concerned that the safe sex messages are not reaching the right people, they have asked for some research to be carried out. They linked information from medical records with information about what benefits a young person or their family received. They wanted to see if wealth influenced teen pregnancies.
Scenario 2: Researchers wanted to know if birth weight and living conditions were related to risk of getting heart disease in later life. They used about 15000 birth records and linked them up with other information, including death records. About 3000 people had already died by the time the study started. Results showed that low birth weight is linked to high blood pressure, increased risk of diabetes, and reduced bone strength. This study showed that risks to babies during pregnancy can affect health later in life.
Scenario 3: Recent research has shown that people with mental health illnesses are more likely get into trouble with the police. Much of this research is based on what people tell researchers. It has been suggested that people don’t always tell researchers if they have mental health illnesses or if they have been in trouble with the police. Researchers want to see if mental health service users were getting the right kind of support. They searched medical records for ones coded to say they had a mental health illness and then looked in police records to see if that person had been in trouble with the police.
Scenario 4: Over the last few decades there has been a rise in the number of young people being diagnosed with asthma. Researchers in Bristol have put together a database of information about the environment. They know how close together buildings are in certain areas and how much traffic there is across Bristol. They now want to map the locations of young people with asthma. To do this they look at GP records and then use the postcodes of the young people with asthma to see that more people get asthma where there is a lot of traffic.

### Analysis

All interview recordings were transcribed verbatim and any potentially identifying information removed. Familiarisation with the data began by reading and discussing the transcripts to compare and begin to categorise the data, and develop a coding framework. Two researchers read all the transcripts (SA, LB) and coded them according to key themes. A purposive small sample of transcripts, chosen to represent a range of views, was coded independently (JM, RC, AB) to check agreement.

Because of the volume and complexity of the data, this paper focuses on the young people’s opinions about consent in relation to the four scenarios. Thematic analysis was undertaken when all data collection was complete, assisted by the Framework approach to data management [20]. A primary chart was created by one researcher (SA) using sections of the original text relating to the issue of consent for the four different scenarios, and streamlined versions of this main chart were produced as the process of summarising and coding the data progressed. Key terms and phrases were retained while repetition and extraneous text were removed. Overarching themes were identified within which similarities and differences were explored. A second researcher (RC) scrutinised the charts and checked the interpretations, which were further considered and agreed by all the authors.

## Results

Interviews were conducted with 55 young people aged 17 to 19 years, of whom 60 % were students and 56 % were female (Table 2). The majority of participants were healthy, White-British and had participated in ALSPAC from birth. There was a spread of IMD scores across the sample.

**Table 2:** Characteristics of interview participants

Category	Number	Percent
All interviewees	55	100
Age		
17 years	12	21.8
18 years	35	63.6
19 years	8	14.5
Gender		
Female	31	56.4
Male	24	43.6
Ethnicity		
White British	51	92.7
Other	3	5.5
Refused	1	1.8
Qualifications		
At university	7	12.7
A-levels	25	45.5
GCSE’s	8	14.5
Other (e.g. NVQ, BTEC)	12	12.8
None	3	5.45
Employment status		
Employed	10	18.2
Student	36	65.5
Unemployed	9	16.4
Health		
Disability/long term illness	9	16.4
No disability/long term illness	46	83.6
ALSPAC participation history		
Engaged	41	74.5
Partially engaged	4	7.3
Disengaged	6	10.9
Eligible to participate	4	7.3
Index of Multiple Deprivation (IMD) score		
(most deprived) 1	12	21.8
2	16	29.1
3	6	10.9
4	7	12.7
(least deprived) 5	14	25.5

The scenarios are considered in turn below, and the key issues raised are illustrated with quotations. Despite a range of consent procedures being outlined and discussed, the young people tended to consider consent as opt-in consent through which participants are given information about the individual study and specifically ‘asked’ if their data can be used. Table 3 illustrates the broad spread of opinions, by gender, about whether participant consent should be requested for data linkage. This table illustrates that there was no consensus for any of the scenarios: for each of the proposed studies there were young people who said consent was not needed, others who were unsure, and others who felt consent was required.

**Table 3:** Should consent be requested for data linkage?

**1. Linking teenage pregnancy and state benefits**		**2. Linking birthweight and future health outcomes**	
No consent required *n* = 9 (4f, 5 m)	Request consent *n* = 34 (20f, 14 m)	No consent required *n* = 11 (5f, 6 m)	Request consent *n* = 36 (20f, 16 m)
Unclear/unsure *n* = 11 (6f, 5 m)	Study should not take place *n* = 1 (1f)	Unclear/unsure *n* = 8 (6f, 2 m)	
**3. Linking mental health and criminal records**		**4. Linking asthma and postcodes**	
No consent required *n* = 20 (9f, 11 m)	Request consent *n* = 20 (15f, 5 m)	No consent required *n* = 14 (8f, 6 m)	Request consent *n* = 26 (16f, 10 m)
Unclear/unsure *n* = 15 (7f, 8 m)		Unclear/unsure *n* = 15 (7f, 8 m)	

f = female, m = male; total participants = 55

Bold data are the subheadings

### Scenario 1. Linking teenage pregnancy and state benefits

Scenario 1 proposed linking medical records, specifically pregnancy, with records relating to state benefits (financial assistance provided by the government to low income families). Opinions ranged from suggesting the study should not take place at all, through varying emphases on the importance of consent to assertions that no consent was necessary. The potential to offend or stigmatise those on lower incomes led one young woman to reject the idea of the study altogether:

If they’re concerned that safe sex messages aren’t getting through I don’t see how finding out if wealth influenced it is going to make any difference … people would be offended if they even did the study … It’s like stereotyping them … they could just send out more messages overall. *Female, ID4.*

This was the first scenario discussed with respondents and overall there was a strong sense that people had a right to know if researchers were accessing and linking potentially sensitive data about them:

I wouldn’t like to think that someone’s, they’ve based information on people’s medical records and they don’t know that they’ve been used for this survey … it’s not really right to just take this sort of information without asking for it. *Female, ID26*.

Everyone’s got their right to say yes or no, surely … people should still have the right to say “No I don’t want you to access that”, than you going straight in and getting that information. *Male, ID18*.

Others expressed concern about being judged or labelled, and felt the solution was to ensure that consent was given:

I reckon if they’re going to use people’s like, medical document and benefit documents, they should ask the permission of the people they’re going to use … I think they’d be like a bit embarrassed, or a bit like mmm don’t like want that information shared, so she should have the right to say no. So they should be contacted… say I was like 15 and pregnant I wouldn’t want my name to be given because, I don’t know, being judged. (*Female, ID22*)

Just because they could be in low income doesn’t make a difference about their medical records … It’s like putting like a label on them … I would want to be asked if I was being labelled … If they can’t get it [*consent*] then it shouldn’t happen. (*Female, ID8*)

These quotations suggest the perceived importance of consent because of a sense that participants are being singled out, or individually identified by those doing the linkage. For others, such consent should be requested because data about an individual were seen as being owned by that person, not by the agency holding the information, and therefore permission for a third party to use it was required.

Because it’s their information, you know what I mean. They should be asked, it’s their’s and they should be able to say who gets it. (*Male ID30*)

However, because the research involved numerical analyses at an aggregate and therefore ‘impersonal’ level, rather than an individual level, others felt consent may not be required.

It’s medical and benefit records, so it is, you know, private so they probably should ask for consent … I’d like to think someone would ask me but if-something like this where it’s kind of more, not random, but they’re not looking at individual case studies, they’re just after a correlation-I think in that case it probably would be ok to go ahead without specific consent … if you’re just after a correlation then I think that’s quite an impersonal thing anyway. (*Female, ID37*)

Anonymising the data was seen as a solution to concerns about privacy, but for some respondents this did not necessarily remove the need for consent:

Finance is quite a personal subject … I’m not too worried about my financial information but some people might get funny about it so you have to ask them first … So this could be a bit personal, in the medical side of things … There should still be consent even if it is anonymous. (*Male, ID40*)

It can be very personal information, as in pregnancies. Some people might not want you to know they were pregnant or something. Um and then, and state benefits as well, they might not want to talk about that … I think as long as the research is anonymous then it should take priority over consent. (*Male, ID17*)

The following quotation illustrates how some participants moved from one opinion to another as they considered whether anonymisation could remove concerns about using an individual’s data without their knowledge.

They should ask the people that are pregnant … because it’s their information isn’t it, and you'd want to know why their information was being used … like they can choose to give the researchers their medical records and financial details … if it’s not doing it on an individual basis then … I don’t think they should need to be asked… it’s not the individual, it’s a number at that point isn’t it, you’re not looking at the person … It’s not like it’s going to get published in a journal with everyone’s names. (*Female, ID19*)

### Scenario 2. Linking birth weight and future health outcomes

The second scenario proposed accessing medical records to retrospectively link birth weight with future health outcomes including diabetes and heart disease. Seven young people specifically stated that the study proposed in this scenario was more ‘important’ than that in the first scenario, with the benefit to the public more obvious than in the previous scenario.

No I wouldn’t ask for permission if it was me … Well it’s more, it is life and death isn’t it, so it’s not necessarily a young kid getting pregnant. It’s, I think there’s more to gain out of doing that one than there is worrying about teenage pregnancy. (*Male, ID22*)

It’s gonna help people’s health. It’s gonna save money. It’s gonna be less drain on resources, and if you can prevent it or know what to do through childhood then it’s gonna help in the long run … In many ways it would probably be better not to ask for consent. Let’s just go ahead and do it because if you’re gonna base future research on it and you want your data to be accurate, otherwise your gonna come up with some theory that is either pointless or would mean you putting in place things that are actually gonna hinder people as opposed to improve their health, so I’d probably go ahead and do it. (*Female, ID37*)

While some respondents felt the requirement for individual opt-in consent might be waived if there was evidence that linkage was for the public good, others argued it was important to gain consent.

It’s kind of about life and death really … it’s about, you know, discovering why … I still think it should be asked actually. (*Female, ID49*)

I can see both ends of the argument though and that’s why it’s hard to do it, cos you think well yeah you should cos if it’s a good cause you should just let them do it. But then again it’s kind of your information and if you can’t even withhold, withhold the privacy of your information, then what have you got? Like what? We might as well just like plaster it all over your walls and stuff, let everyone see it. (*Male, ID52*)

Although the researcher highlighted some of the practical difficulties, time and expense involved in attempting to contact 15,000 people, this did not appear sufficient to change strongly held opinions about whether consent was needed.

Consent should always be asked … I think, however expensive it is, some sort of consent needs to be asked for. (*Female, ID11*)

Then researchers would just make their research with big groups so they didn’t have to ask permission so it would just be an escape, an escape route really, wouldn’t it … I probably wouldn’t want to take legal action or anything but I’d be offended [*laughs*] that they’ve just not asked me. (*Male, ID2*)

You should always be asked no matter how many people it is. (*Male, ID34*)

Yeah [*consent needed even if expensive to obtain*] cos if the research is really important, then it should get funding from the government. (*Female, ID42*)

A further important point of discussion was whether consent was required to access the data of those who had died. Opinions ranged from asserting that these data should not be used at all, through considering whether opt-out consent had already been given or family members should be consulted, to suggesting the data could be accessed without any consent.

Well you can’t get permission from someone who’s dead. So I don’t think that they should use any data of people that have already died … No, I don’t like the fact that like you can’t give consent. (*Female, ID28*)

They might have said you could use it before they died [*opt-out consent*] … if not then maybe if the family agrees … They’re not really going to know whether information’s being used but still you shouldn’t, you shouldn’t just be able to, somebody just use it without any kind of consent at all. (*Male, ID47*)

I think you probably have to have permission from their relatives and if they haven’t got any relatives then I would just use it. (*Female, ID51*)

Sometimes like dead people’s information can be like the best information because they’ve already had a bad out-a bad outcome, and you can find out why, and that can be like the most important stuff … Just take the information if it’s, if it’s really important, then just take it, who’s going to care because they won’t know? (*Male, ID12*)

The suggestion that no form of consent is needed in order to gain ‘really important’ information, relates to the preference for research that is for the ‘greater good’.

### Scenario 3. Linking mental health and criminal records

The third scenario proposed linking mental health and criminal records. Discussions of this scenario were complicated by uncertainty about whether people with mental health problems would have the capacity to give informed consent.

Big mental health illness, they probably won’t be able to like make their own decisions anyway. (*Female, ID51*)

The only thing I’m slightly thinking about is if people with mental health illnesses would they be able to understand … it’s all such a very big category isn’t it mental health illness. You’d have to look at different types of mental health illness I suppose. (*Male, ID24*)

The purpose of this research was mentioned by young people who asserted that opt-in consent should be sought, and those who felt it was not required:

I don’t see what you can get from the information once it’s collected because quite often that those sort of correlations have already been spotted in society … I will always say consent … I think you should ask consent of the individual. (*Male, ID45*)

I think it’s fine just to do it … I think because it says like it wants to see if the mental health service users are getting the right kind of support, it’s important that they do have a right to sort of support … I think that’s OK because again it’s just like trying to help them, but it’s not just for pointless, just general knowledge. (*Female ID27*)

Concerns about the purpose of the research were closely linked to the stigma associated with mental illness:

That’s kind of labelling and stereotyping those mental health people, and it’s quite wrong to pin-point those certain people really … Yeah, definitely will need consent cos at the end of the day it’s going to be published out and, and people would want to know what is going to be used from their records really. (*Female, ID23*)

There is still a lot of stigma attached for mental health … they probably should [*seek opt-in consent*] for this because it is, you know it’s, somebody might find out they’ve got mental illness and they don’t want them to know … you’d have to look at the positive, look at the negative and if the positives outweigh the negatives perhaps don’t ask them their permission. (*Female, ID1*)

Despite the researcher explaining the processes for anonymisation in some detail during the earlier part of the interviews, many of the young people’s comments seem to be predicated on an understanding that people’s identity may be revealed. This is suggestive of a lack of trust of how the information would be handled in the research and concerns about the limits of anonymisation. Consequently this scenario elicited responses, especially from young women, about the ethics of the research and the potential for harm:

It seems like it could cause prejudices against people with mental illnesses and say like they’ll all, all go out and commit crimes or something … I guess people’s information being used behind their back, so they don’t know that they, they’re part of the study … might stop some people from um like going to the doctor when they need to or something cos they don’t want it to be put on their medical records. (*Female, ID42*)

People shouldn’t be looking through medical records going “This person has a mental illness”. Like that’s really, that’s so unethical … Well I actually do have a mental health illness and I wouldn’t want people looking at my like records going “Oh you know she’s like mentally ill” … I know people like need more help and stuff and like it’s good to research like the kinds of support and that sort of stuff, but it’s definitely something that I think needs informed consent especially when police records are involved as well. (*Female, ID28*)

This one does seem like really quite invasive I think … in some weird way, it feels like some sort of persecution type of thing. I mean it’s not, but it does sort of seem a little like that … I feel like the research is really important but um, maybe it’s kind of um, permission should definitely just be asked for it … It just seems like an easy option to just be like “Oh it doesn’t matter” and just sort of say “Oh they can link those two, it doesn’t, they’re not gonna know, it’s not gonna personally affect them, it’s for the greater good”. But then, and I’m not sure, I mean a lot of things are for the greater good and they’re not all that good themselves. (*Female, ID33*)

Amongst the respondents, some young men focussed on the issue of not gaining accurate information and the potential for bias:

It says on here “people might not tell researchers they have mental illness” … I guess you could just take the information … because it makes it more accurate and reliable. (*Male ID12*)

Wouldn’t be much use by asking because as it says somewhere that people that have been in trouble with the police and have mental illness don’t tend to tell people, so asking consent you’d get all the people who haven’t been in trouble … so don’t get consent … just do it. (*Male, ID17*)

If they’ve got a mental health illness then they may be more likely, that might affect their willingness, so it might be hard to um actually gather the right, gather enough information. I think that might be biased … maybe you have to, not have to, use it without their permission in order to help, because you’re going to help them overall really in the end. (*Male ID47*)

This scenario also prompted discussion about the importance of anonymising data which may mitigate the requirement for consent:

There is certain situations where you might be able to, it might be acceptable to ask or it might be acceptable just to go ahead and get it … as long as it wasn’t directly linked back to you, as a person, it would be all right … So it’s just like a list rather than actual people. (*Female, ID6*)

I think this one’s complicated, as to whether or not um you’d have to ask them. I think probably not … as long as the information about what sort of mental health thing, and what sort of get in trouble with the police, was not so detailed that you could get back to them. (*Male, ID24*)

If the system actually managed to work and it wasn’t traceable back to anyone at any point really then it would be all right. It’s just things that could go wrong with it I suppose … someone gets access to the data whilst there’s still names attached. (*Male, ID32*)

That’s quite confidential though, mental health one, this is and then looking at their criminal records as well … You’d have to ask them, but I wouldn’t be very happy with it, I tell ya … If it was anonymous then I suppose it could be all right. (*Male, ID40*)

### Scenario 4. Linking asthma and postcodes

The fourth and final scenario proposed to map the locations of people with asthma which would require accessing their medical records and obtaining their postcodes. Amongst the young people interviewed, there was a predominant opinion that asthma is not a stigmatising condition. However, this did not result in a consistent response about whether consent should be requested for this study:

It’s not making anyone shy away, it’s literally just if you’ve got asthma. Everyone’s going to know you’ve got asthma because you’re going to be like, got an inhaler … It’s just ethically right to do it [*gain opt-in consent*] you know, it’s the fair thing to do and it’s their information at the end of the day. (*Male, ID30*)

It’s not as sensitive as the others … I don’t imagine that a lot of people are ashamed because they’ve got asthma, so that I probably think that people would be OK and want to know why they’ve got asthma … I’d probably say you could ask consent. I don’t think it would matter either way because in the long run it would benefit them. (*Female, ID38*)

I suppose, in a way, asthma seems like a relatively harmless thing to look at GP records for but I don’t know. I feel like that shouldn’t really bias me but I mean, addresses kind of seems like the, slightly invasive … I mean people have like wildly different sort of opinions … like my parent’s generation would be kind of like, you know, mental health thing would be a thing to sort of hide and sort of, maybe a bit sort of shame or something. Or, or, you know, a sort of pregnancy or something that you didn’t want would be like that as well, um, and they might not want people to have access to, you know. Some people still sort of have that opinion. Whereas others really don’t mind and I think it’s just important to have that sort of choice. But, yeah, for me, asthma’s like not a big deal … I think it would be best if people did have their permission asked, but um I don’t know, it doesn’t seem like it would be that horrendous if it wasn’t. (*Female, ID33*)

The requirement for opt-in consent appeared more closely associated with using participants’ postcodes which were seen as more specific to the individual.

I think it wouldn’t be about the asthma, it would be about doing a thing about where you live. (*Female, ID6*)

They definitely need consent with that one ‘cos it’s, ‘cos they keep addresses. Certain, it’s like locating people and they might not want to have everyone knowing where they’re, where they live sort of thing, like the researchers and looking at GP records they might be a bit sensitive ‘cos they could look at anything on the GP records. (*Female, ID43*)

If it was the whole postcode then that’s quite confidential ‘cos you’re giving away where someone lives then … the researchers have to have the details, as long as they don’t abuse it I suppose … Like using it for wrong purposes, giving it on to third parties. (*Male, ID40*)

Anonymising and ensuring security of the data were identified as solutions to the potential invasion of privacy:

So as long as it stays within, you know, where it’s got to be then I think that would be all right, but as long as it doesn’t, you know, information doesn’t accidentally get out … if all their information’s getting thrown away and it is just a number of like how built up the area is or something then no I don’t, don’t think it would be a problem. (*Female, ID13*)

As it becomes like less anonymous I think you should act like, consider consent more … I don’t know like if there’s a lot of people with asthma or not so it, it depends like what numbers because there could be like hundreds or thousands of people with asthma. So it could become like really difficult to get consent … I think if numbers are like really large then I don’t think you need to worry about consent with this one. (*Male, ID35*)

I don’t see why you have to ask consent if there is, if it is made anonymous … the admin staff know who you are, it can be still can be traced back … I think yes you should ask for consent but if it is going to be completely anonymous, and the governing bodies decide that it is gonna be anonymous, then I don’t see any reasonable reason why you should have to ask for consent. (*Male, ID45*)

The following quotation illustrates an attempt to balance a perceived invasion of privacy against the needs and purpose of the research.

It’s not the most personal illness ever, and in that case I think that it [*data*] should just be taken. But I think essentially you’ve got to keep the same for everything because you just lower the line and eventually every situation doesn’t require permission. So I would say that they’ve got to ask for permission … The main cost is only a little bit of invasion of privacy and I guess that is kind of, that’s kind of overshadowed by the fact that it is a beneficial survey. It’s not like they are trying to find ways in order to hurt the people with asthma, they are finding ways to help them. (*Male, ID5*)

This scenario also prompted assertions that requiring opt-in consent was unlikely to undermine the research because such consent was likely to be given:

I think if you asked those people they wouldn’t mind anyway … if you asked, everyone would, not everyone, but majority of people would say yeah, and so might as well ask when you can. (*Female, ID4*)

It’s fine because you’re not, it’s not like you’re labelling them with something. It’s like a normal thing … Just ask them, just so they wouldn’t, just so they know that their address will, might, their address would be on a map. But knowing, I don’t think they would have a problem with it, cos it’s only just saying where they’ve got asthma to, and how close they all are. (*Female, ID8*)

I think they‘ll be more willing to like say “Yeah” or “Do it” kind of thing, or it will be their mums or whatever, but like I reckon they’ll be more willing to. Cos like if it’s their mums or something they’ll be like “Oh it will help kids?” and stuff, and mums like doing that I guess [*laughs*]. (*Female, ID25*)

## Discussion

### Public attitudes to data linkage

The issues identified in this study with young people resonate with those of previous research eliciting opinions about data linkage from a wider age-range. Qualitative research commissioned by Wellcome Trust in 2013 examined public attitudes to linking personal data. Focus groups and telephone interviews were undertaken with 50 men and woman aged 18–70 years from different socio-economic groups [21]. The research concluded health data may be perceived as different from other kinds of data: although health data were regarded as personal and private, it appeared acceptable to share these data within a trusted medical context. There were very few objections to medical data being used for the ‘general good’ (such as helping to find cures and causes of disease) as long as commercial gain was not the priority. Some awareness of data linkage by government departments was evident, with a perceived benefit of identifying fraudsters. Nevertheless, anonymity and consent were considered important and there was some unease about individuals being targeted and ‘blamed’, especially amongst respondents with lower socio-economic status. The researchers concluded that the public were not particularly sensitive about research that involves linking health and other data, providing the objective is to increase knowledge around the causes and cures of ill health.

In 2011 the Welsh Government commissioned research to test a new Welsh Health Survey (WHS) procedure seeking consent from participating individuals to link their survey answers with other information held about them elsewhere [22]. Respondents’ reasons for giving consent included: wanting to help researchers or be part of improving the health service; having nothing to conceal; believing the data were confidential, and they could withdraw consent if they changed their mind, and; contributing to a ‘bigger picture’ that could lead to more worthwhile results. However, there were some concerns including: not feeling confident that they had understood the form; not wanting to give researchers ‘carte blanche’ to do whatever they wished, and; lack of clarity about what could be included under ‘social and lifestyle’ data, who could have access to this data and what it would be used for. In March 2013 a further report was published about the Welsh Health Survey, and the request to link data, which indicated that 59 % of respondents gave permission for data linkage (of whom 96 % provided the full name and date of birth, and 4 % provided full name but no date of birth) [23]. A similar proportion of men and women consented to data linkage with no difference by socio-economic status, but younger adults were marginally less likely to consent.

A Scottish Government study in 2012 examined attitudes to a proposed Data Linkage Framework during three workshops with a total of 73 people [24]. The authors reported most participants were positive about data linkage as long as it was not ‘abused’ by public bodies or individuals. Participants felt a minimum requirement of research involving data linkage should be that it is 'in the public interest' which was defined in terms of tangible benefits such as medical advancements or improved services. There was a consensus that procedures and processes surrounding data linkage should be clear and transparent. Participants were more positive about the idea of data linkage after being informed that, in most cases, the data would be anonymised. However, some participants, particularly those in the youngest age-band (18–34 years), said they were not concerned about the use of named data provided they were not contacted and their data was kept secure. They tended to say that they had "nothing to hide" and so had no qualms about their personal details being used in research.

An Australian study exploring lay people’s views of data linkage involved in-depth interviews with 26 participants [25]. Participants were provided with information regarding best practice data linkage processes through discussion and diagrams, and four hypothetical data linkage scenarios of increasing complexity were discussed. The author concluded that lay people have the capacity to understand data linkage and anonymisation processes and, while privacy protection remained an important consideration, the level of protection afforded in best practice data linkage was viewed by most participants as sufficient protection for data linkage to proceed without specific individual consent.

### Key findings from the scenarios in the PEARL interviews

Overall the participants held positive views about health research and believed it was important to participate in studies for the wider public good. The scenarios relating to teenage pregnancy and mental health elicited unease about individuals being stigmatised or blamed, while scenarios relating to heart disease and asthma tended to be seen as having a clearer purpose and health outcome. Thus the young people’s comments support other findings which stated a preference for research with tangible health benefits and a willingness to contribute to research which was for the general good.

Despite a range of consent procedures being explained, the young people tended to equate consent with opt-in consent through which participants are given information about a study and specifically ‘asked’ if their data can be used. This is perhaps not surprising. In everyday situations consent is usually equated with giving permission rather than the absence of a refusal. This would suggest that providing information, and allowing time for discussion, may be insufficient to change perceptions of what consent ‘really’ means. Anonymisation procedures were also explained but this was not always seen as sufficient to eliminate the requirement for consent: there was some evidence of a lack of trust about how data would be used and concerns about the limits of anonymization. There was also evidence that for some people disclosure risk was not the only issue. Even where this risk could be effectively mitigated through anonymization some individuals still felt it polite that their permission should be sought for secondary use of information they perceived they ‘owned’. Together these findings suggest that greater public discussion is needed about the benefits of data linkage for health research, the processes involved to protect participants, and how to balance the ‘rights’ of individuals to prevent the secondary use of their personal information with the ‘responsibilities’ of allowing such use in the public interest when reasonable steps have been taken to protect their identity.

Three-quarters of the PEARL interview participants had participated in regular ALSPAC assessments throughout their lives. ALSPAC has communicated regularly with the cohort and there is a website giving information about the research studies involved and the important findings to date (http://www.alspac.bris.ac.uk). This is likely to have built up a degree of understanding of the research process, and trust in the integrity of the research team, that would not be found in a more general sample of young people. However, even with potentially high levels of trust, there was a spread of opinion about the use of personal or sensitive data and consent requirements. Opinions ranged from asserting that opt-in consent should always be sought to suggesting that in some cases it might be best if researchers just got on and used the data they needed. Broadly categorising the young people’s opinions into ‘no consent required’, ‘request consent’ and ‘unclear/unsure’ illustrates that there was no scenario for which a majority of interview participants asserted that requests for consent were not needed. This appeared to be linked to notions of ‘ownership’ that individuals feel for information that relates to them, as well as a lack of clarity about the extent to which ‘personal’ data would or could be de-identified.

The threat to privacy, though sharing the ‘personal’ information of study participants without their knowledge or consent, was identified by participants as a potential harm. However, the definition of ‘personal’ varied between young people. An address was considered personal by some, and less so by others because it was in the public domain. Medical conditions such as pregnancy and mental health illness were likely to be considered personal, but asthma less so. These definitions were influenced by whether the interviewee associated stigma with the condition or data required, which might be a medical condition, a criminal record or poor neighbourhood.

In many cases the young people did not simply consider their own opinions but acknowledged that different people held different views. Consequently, although they themselves had an opinion about being asked for consent, they could not speak for others. The quotations illustrate that the time and expense involved in gaining sufficient participants for studies that need large numbers may be accepted as a justification for forgoing consent, or considered an ‘escape route’ for researchers that should not be used to deny potential participants their right to decide whether their data would be used for research purposes.

For some young people, their belief that most people would *not* object to a study taking place was not a reason to forego seeking consent. Rather they felt consent should be sought as a means of keeping people informed about the use of their data whilst not undermining the research process. This was also linked to etiquette: there was a suggestion that it was ‘fair’ and ‘right’ to ask for someone’s permission before using their data. This view is compatible with, for example, the UK Medical Research Council guidance that it is always best practice to obtain consent wherever this is practicable [26].

A noticeable finding from this study is that young people raising the same issues came to different conclusions about whether consent was needed. For example, in relation to the teenage pregnancy scenario, one quotation illustrates the belief that anonymising the data was sufficient to prioritise the research needs over consent, while another asserts that consent should be requested even if the data were anonymised. Similarly, quotations from young people who considered that the study linking birth weight and heart disease was important show that one concludes “I wouldn’t ask permission” while another asserts consent should be sought even if it is difficult to attain.

The sensitivity of personal data, and anticipated benefits of research for the wider public good, were identified by the young people as two important factors to be taken into consideration when deciding whether opt-in consent was needed. However, there was no clear pattern to suggest that one of these factors was more important than the other for a majority of participants. Rather, young people varied in the way they defined and balanced these issues, and this could change for a given individual in relation to the different scenarios. This reinforces the finding that there is no ‘one size fits all’ solution to the complexity of decision-making in this field.

Some of the findings here resonate with findings about consent in other areas of health research that question the extent to which, despite being given information, people fully understand what they are consenting to [27, 28]. Williams et al. suggest that, as well as focusing on the information and discussions needed to give informed consent, it is important to consider what potential participants need to know to provide an informed refusal [29]. This debate can be broadened further to include whether people fully understand different types of consent. Our evidence suggests that even individuals with a long history of involvement in a research project (including repeated exposure to consent procedures) show evidence of uncertainty and inconsistency around these issues. This may call into question the validity of focussing on ostensible individual consent as the main consideration in relation to the secondary use of personal data [30].

### Strengths and limitations

This study involved a relatively large sample size in order to increase the diversity of participants. Data saturation was reached with no new issues being raised by additional interviews. However, most of those interviewed for the PEARL study are long-term participants in ALSPAC and their understanding of research is unlikely to be typical of young people who are not in the ALSPAC cohort. Nevertheless, a wide range of views were expressed. While it is not possible to generalise from the results of this or any qualitative study, the opinions expressed are authentic and raise interesting issues.

Although the researcher explained key concepts and procedures, and discussion was aided by scenarios, the issue of informed consent is complex. Different types of consent and anonymisation strategies were explained but some misunderstandings were apparent and this affected young people’s ability to discuss the scenarios in a knowledgeable way. However, this lack of clarity is likely to be reflected in the wider population.

## Conclusion

For many participants in this qualitative study, the requirement for consent appeared to be based on the individual research proposal and the sensitivity of the personal information involved. This appeared in turn to be related to concerns around the potential for harm from the disclosure of identifiable sensitive information. For some individuals these concerns could apparently be mitigated through anonymisation of the data. Others appeared to either not understand, or not believe, that effective anonymisation was possible; or expressed a view that, even where personal data were effectively anonymised, consent should be sought for reasons of etiquette and trust, as well as a sense of personal ownership. Despite different forms of consent being explained, participants tended to focus on the importance of informed opt-in consent. However, some individuals adopted apparently contradictory positions during the course of a single interview. Public views on the issue of consent in relation to linkage based research are clearly complex and diverse. Accommodating these views within a governance framework that is acceptable to all or even a majority of the public is likely to be challenging. If research using data linkage is to successfully realise its potential for public good without undermining public trust in the research process then pragmatic, imaginative and flexible approaches to these issues are needed.

## Abbreviations

ALSPAC: avon longitudinal study of parents and children
IMD: index of multiple deprivation
NHS: National Health Service
PEARL: Project to Enhance ALSPAC through Record Linkage
